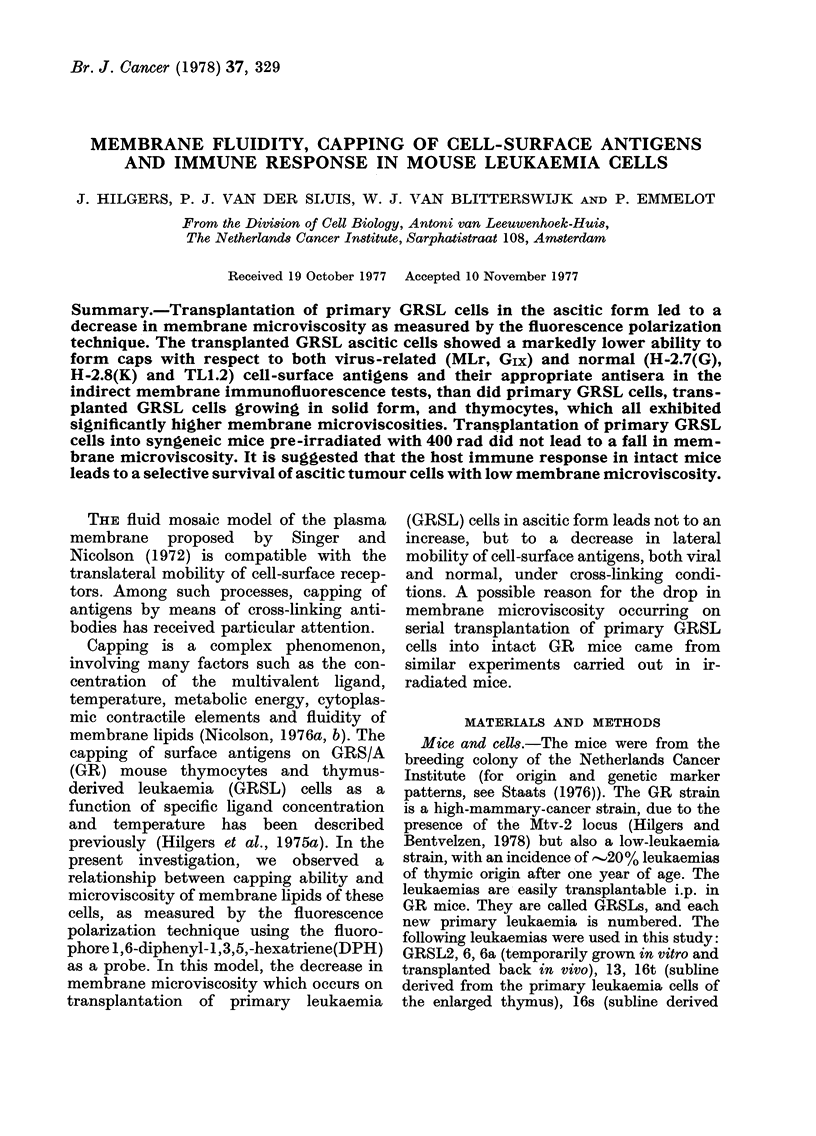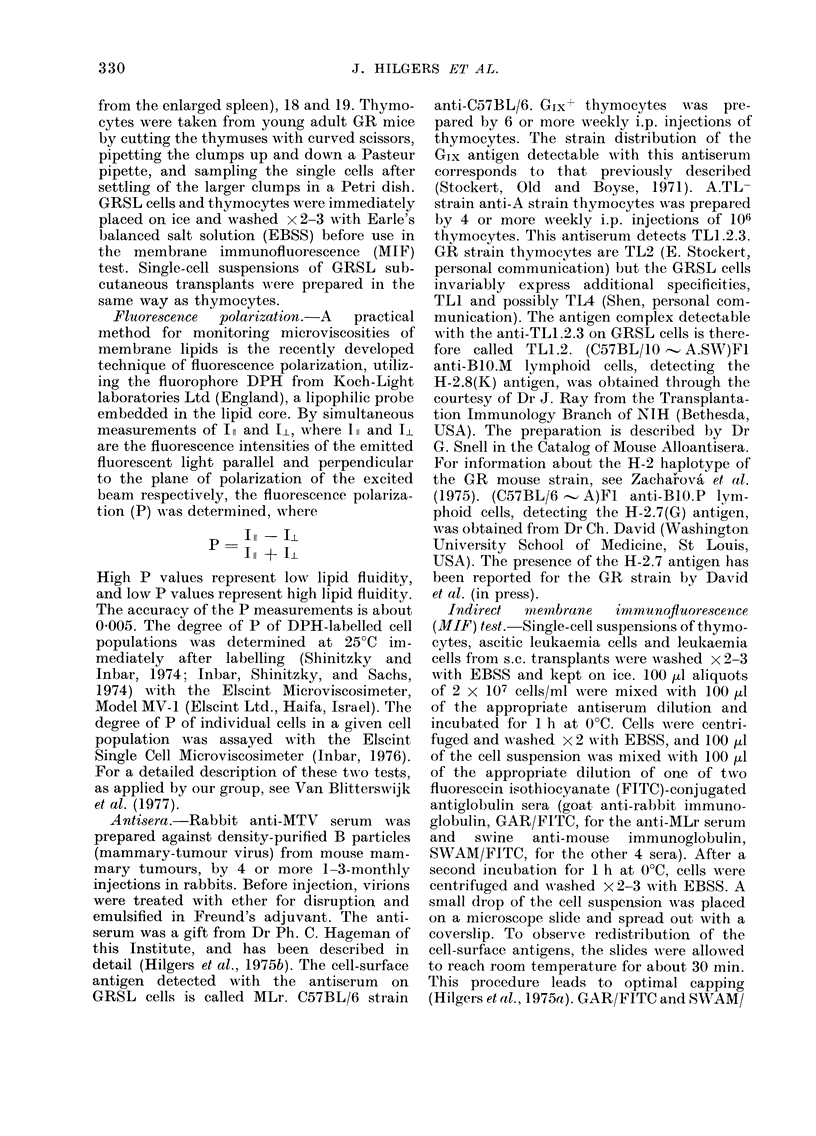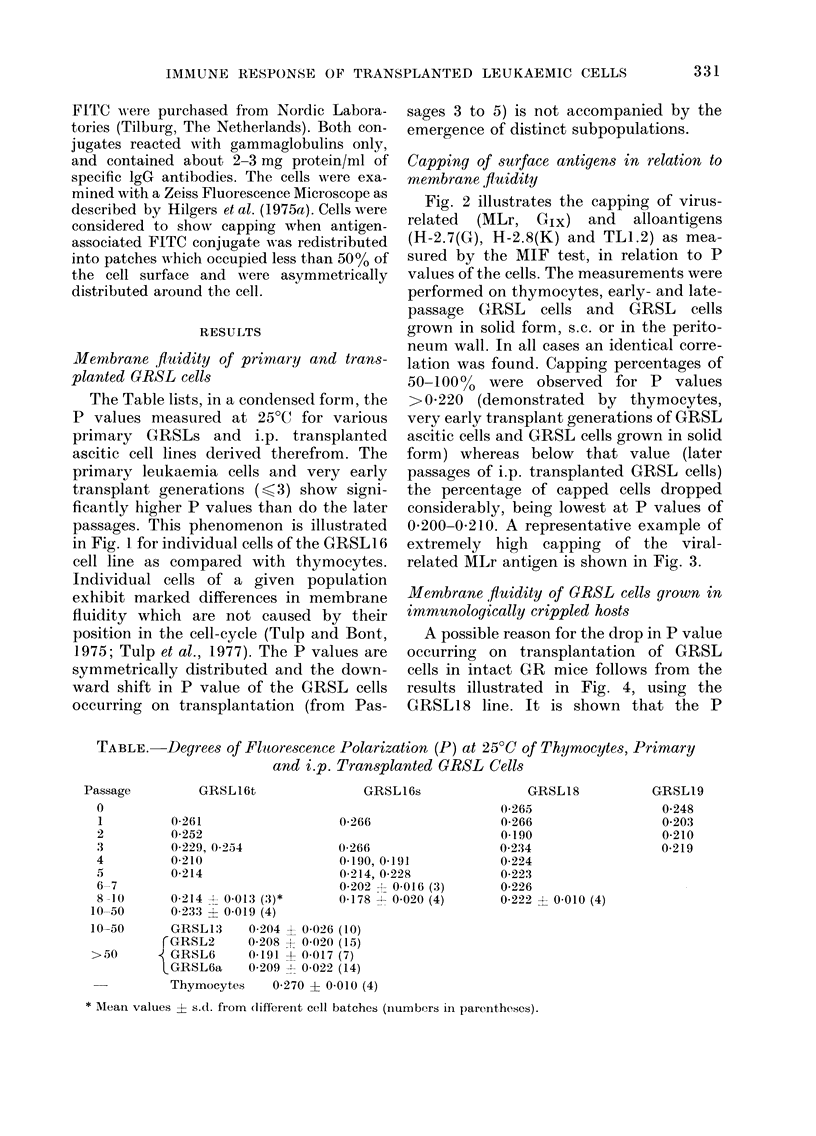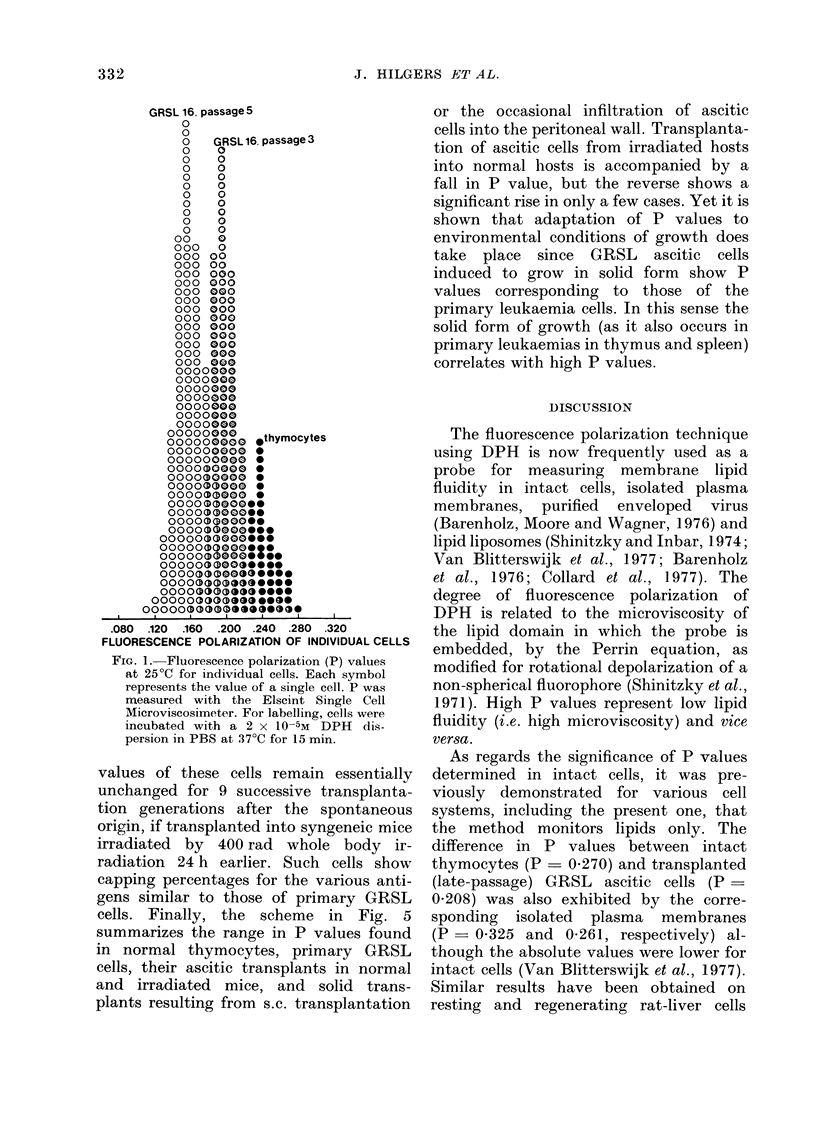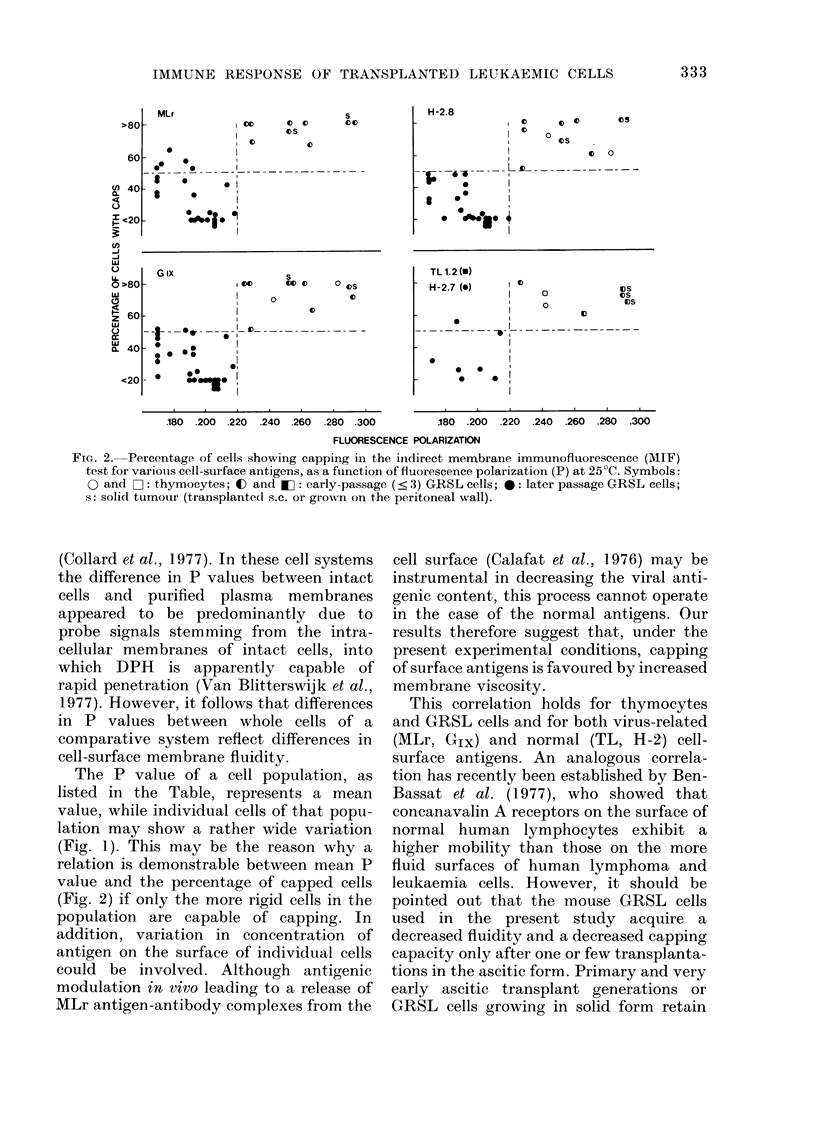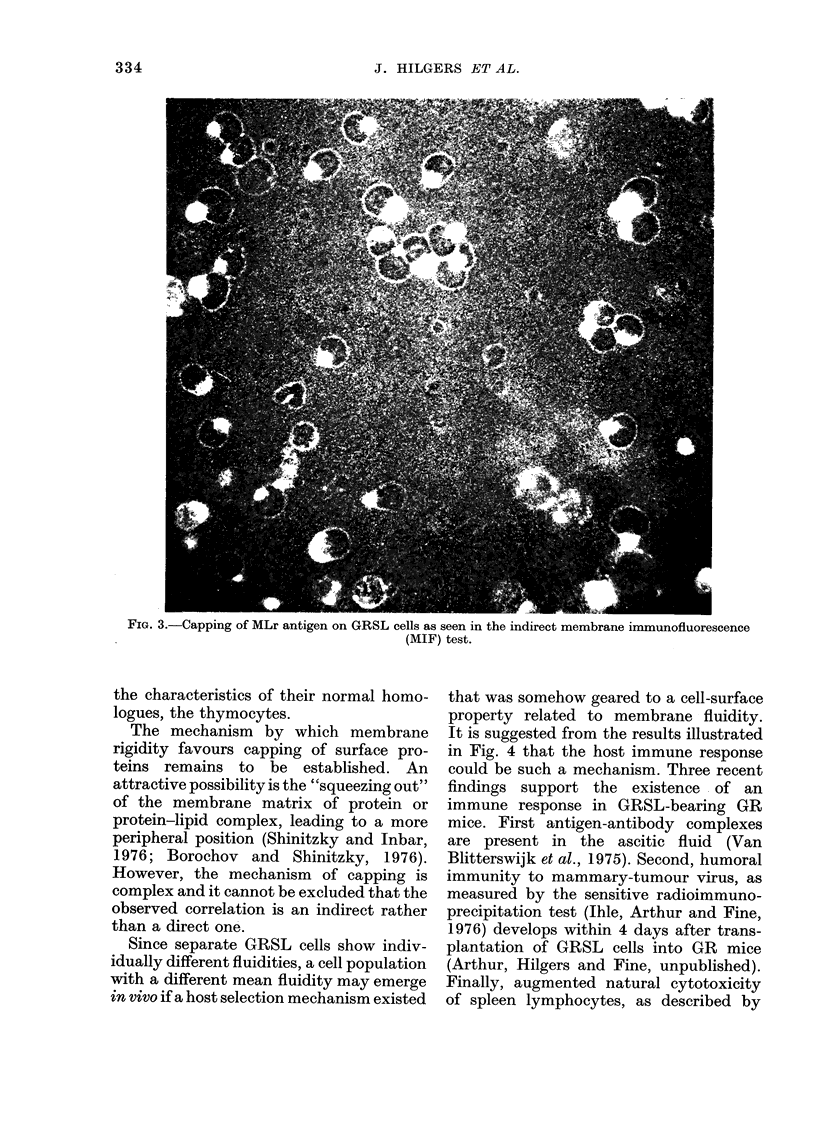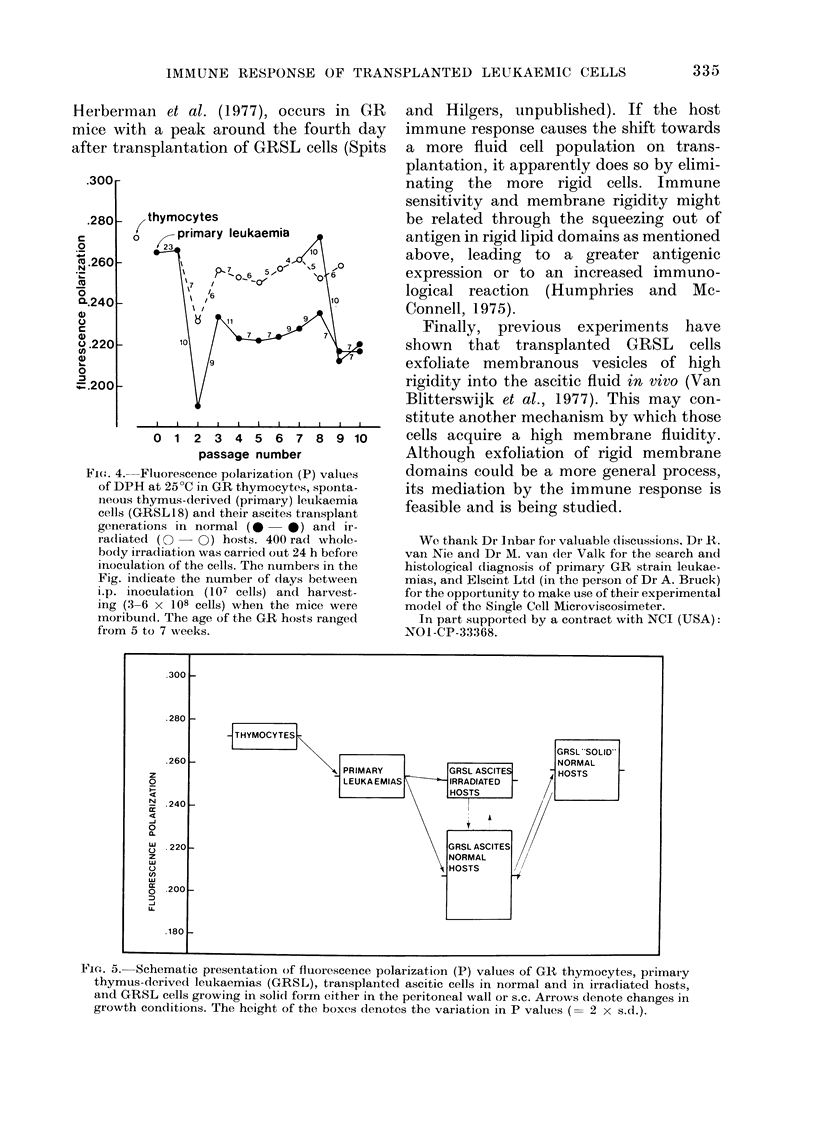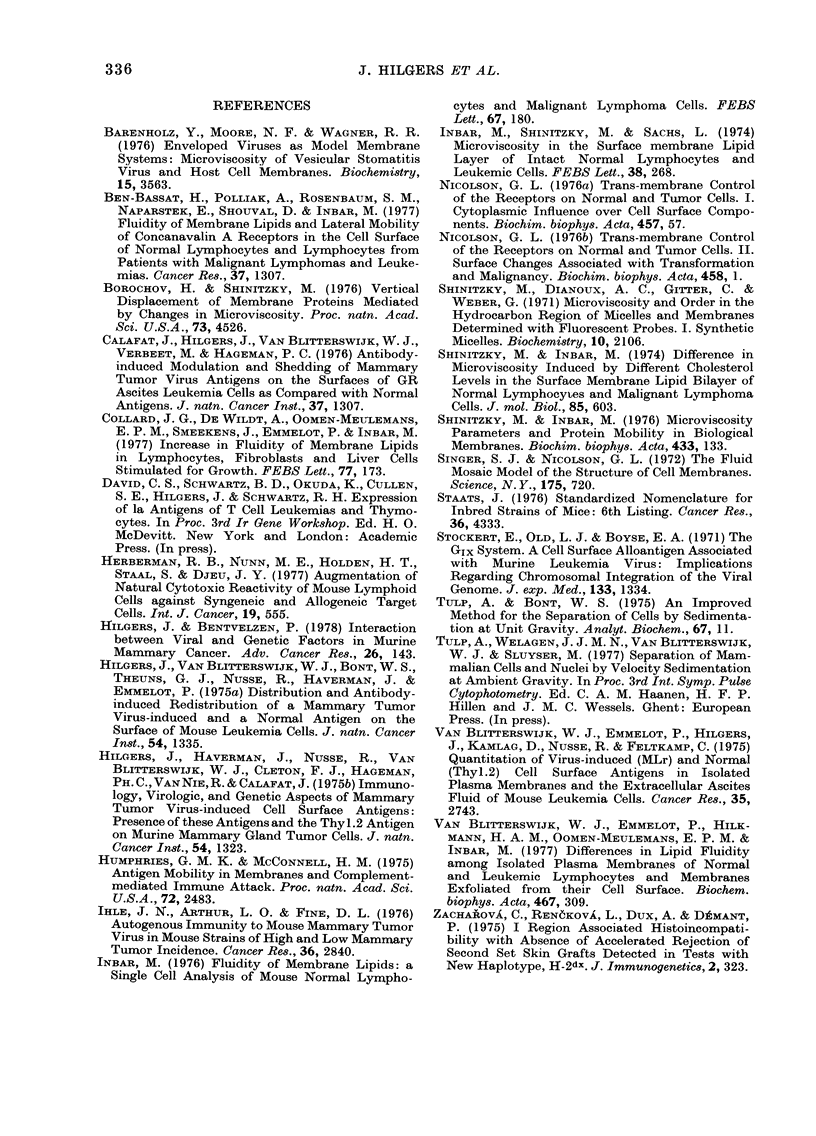# Membrane fluidity, capping of cell-surface antigens and immune response in mouse leukaemia cells.

**DOI:** 10.1038/bjc.1978.51

**Published:** 1978-03

**Authors:** J. Hilgers, P. J. van der Sluis, W. J. van Blitterswijk, P. Emmelot

## Abstract

**Images:**


*Br. J. Cancer* (1978) **37**, 329

### MEMBRANE FLUIDITY, CAPPING OF CELL-SURFACE ANTIGENS AND IMMUNE RESPONSE IN MOUSE LEUKAEMIA CELLS

J. HILGERS, P. J. VAN DER SLUIS, W. J. VAN BLITTERSWIJK AND P. EMMELOT

*From the Division of Cell Biology, Antoni van Leeuwenhoek-Huis, The Netherlands Cancer Institute, Sarphatistraat* 108, *Amsterdam*

Received 19 October 1977 Accepted 10 November 1977

**Summary.—Transplantation of primary GRSL cells in the ascitic form led to a decrease in membrane microviscosity as measured by the fluorescence polarization technique. The transplanted GRSL ascitic cells showed a markedly lower ability to form caps with respect to both virus-related (MLr, $G_{IX}$) and normal (H-2.7(G), H-2.8(K) and TL1.2) cell-surface antigens and their appropriate antisera in the indirect membrane immunofluorescence tests, than did primary GRSL cells, transplanted GRSL cells growing in solid form, and thymocytes, which all exhibited significantly higher membrane microviscosities. Transplantation of primary GRSL cells into syngeneic mice pre-irradiated with 400 rad did not lead to a fall in membrane microviscosity. It is suggested that the host immune response in intact mice leads to a selective survival of ascitic tumour cells with low membrane microviscosity.**

THE fluid mosaic model of the plasma membrane proposed by Singer and Nicolson (1972) is compatible with the translateral mobility of cell-surface receptors. Among such processes, capping of antigens by means of cross-linking antibodies has received particular attention.

Capping is a complex phenomenon, involving many factors such as the concentration of the multivalent ligand, temperature, metabolic energy, cytoplasmic contractile elements and fluidity of membrane lipids (Nicolson, 1976*a*, *b*). The capping of surface antigens on GRS/A (GR) mouse thymocytes and thymus-derived leukaemia (GRSL) cells as a function of specific ligand concentration and temperature has been described previously (Hilgers *et al.*, 1975*a*). In the present investigation, we observed a relationship between capping ability and microviscosity of membrane lipids of these cells, as measured by the fluorescence polarization technique using the fluorophore 1,6-diphenyl-1,3,5,-hexatriene(DPH) as a probe. In this model, the decrease in membrane microviscosity which occurs on transplantation of primary leukaemia (GRSL) cells in ascitic form leads not to an increase, but to a decrease in lateral mobility of cell-surface antigens, both viral and normal, under cross-linking conditions. A possible reason for the drop in membrane microviscosity occurring on serial transplantation of primary GRSL cells into intact GR mice came from similar experiments carried out in irradiated mice.

### MATERIALS AND METHODS

*Mice and cells.*—The mice were from the breeding colony of the Netherlands Cancer Institute (for origin and genetic marker patterns, see Staats (1976)). The GR strain is a high-mammary-cancer strain, due to the presence of the Mtv-2 locus (Hilgers and Bentvelzen, 1978) but also a low-leukaemia strain, with an incidence of ~20% leukaemias of thymic origin after one year of age. The leukaemias are easily transplantable i.p. in GR mice. They are called GRSLs, and each new primary leukaemia is numbered. The following leukaemias were used in this study: GRSL2, 6, 6a (temporarily grown *in vitro* and transplanted back *in vivo*), 13, 16t (subline derived from the primary leukaemia cells of the enlarged thymus), 16s (subline derived from the enlarged spleen), 18 and 19. Thymocytes were taken from young adult GR mice by cutting the thymuses with curved scissors, pipetting the clumps up and down a Pasteur pipette, and sampling the single cells after settling of the larger clumps in a Petri dish. GRSL cells and thymocytes were immediately placed on ice and washed ×2–3 with Earle's balanced salt solution (EBSS) before use in the membrane immunofluorescence (MIF) test. Single-cell suspensions of GRSL subcutaneous transplants were prepared in the same way as thymocytes.

*Fluorescence polarization.*—A practical method for monitoring microviscosities of membrane lipids is the recently developed technique of fluorescence polarization, utilizing the fluorophore DPH from Koch-Light laboratories Ltd (England), a lipophilic probe embedded in the lipid core. By simultaneous measurements of $I_{\parallel}$ and $I_{\perp}$, where $I_{\parallel}$ and $I_{\perp}$ are the fluorescence intensities of the emitted fluorescent light parallel and perpendicular to the plane of polarization of the excited beam respectively, the fluorescence polarization (P) was determined, where

$$P = \frac{I_{\parallel} - I_{\perp}}{I_{\parallel} + I_{\perp}}$$

High P values represent low lipid fluidity, and low P values represent high lipid fluidity. The accuracy of the P measurements is about 0·005. The degree of P of DPH-labelled cell populations was determined at 25°C immediately after labelling (Shinitzky and Inbar, 1974; Inbar, Shinitzky, and Sachs, 1974) with the Elscint Microviscosimeter, Model MV-1 (Elscint Ltd., Haifa, Israel). The degree of P of individual cells in a given cell population was assayed with the Elscint Single Cell Microviscosimeter (Inbar, 1976). For a detailed description of these two tests, as applied by our group, see Van Blitterswijk *et al.* (1977).

*Antisera.*—Rabbit anti-MTV serum was prepared against density-purified B particles (mammary-tumour virus) from mouse mammary tumours, by 4 or more 1–3-monthly injections in rabbits. Before injection, virions were treated with ether for disruption and emulsified in Freund's adjuvant. The antiserum was a gift from Dr Ph. C. Hageman of this Institute, and has been described in detail (Hilgers *et al.*, 1975*b*). The cell-surface antigen detected with the antiserum on GRSL cells is called MLr. C57BL/6 strain anti-C57BL/6. $G_{IX}^{+}$ thymocytes was prepared by 6 or more weekly i.p. injections of thymocytes. The strain distribution of the $G_{IX}$ antigen detectable with this antiserum corresponds to that previously described (Stockert, Old and Boyse, 1971). A.TL$^{-}$ strain anti-A strain thymocytes was prepared by 4 or more weekly i.p. injections of $10^6$ thymocytes. This antiserum detects TL1.2.3. GR strain thymocytes are TL2 (E. Stockert, personal communication) but the GRSL cells invariably express additional specificities, TL1 and possibly TL4 (Shen, personal communication). The antigen complex detectable with the anti-TL1.2.3 on GRSL cells is therefore called TL1.2. (C57BL/10 ∼ A.SW)F1 anti-B10.M lymphoid cells, detecting the H-2.8(K) antigen, was obtained through the courtesy of Dr J. Ray from the Transplantation Immunology Branch of NIH (Bethesda, USA). The preparation is described by Dr G. Snell in the Catalog of Mouse Alloantisera. For information about the H-2 haplotype of the GR mouse strain, see Zachařová *et al.* (1975). (C57BL/6 ∼ A)F1 anti-B10.P lymphoid cells, detecting the H-2.7(G) antigen, was obtained from Dr Ch. David (Washington University School of Medicine, St Louis, USA). The presence of the H-2.7 antigen has been reported for the GR strain by David *et al.* (in press).

*Indirect membrane immunofluorescence (MIF) test.*—Single-cell suspensions of thymocytes, ascitic leukaemia cells and leukaemia cells from s.c. transplants were washed ×2–3 with EBSS and kept on ice. 100 $\mu$l aliquots of $2 \times 10^7$ cells/ml were mixed with 100 $\mu$l of the appropriate antiserum dilution and incubated for 1 h at 0°C. Cells were centrifuged and washed ×2 with EBSS, and 100 $\mu$l of the cell suspension was mixed with 100 $\mu$l of the appropriate dilution of one of two fluorescein isothiocyanate (FITC)-conjugated antiglobulin sera (goat anti-rabbit immunoglobulin, GAR/FITC, for the anti-MLr serum and swine anti-mouse immunoglobulin, SWAM/FITC, for the other 4 sera). After a second incubation for 1 h at 0°C, cells were centrifuged and washed ×2–3 with EBSS. A small drop of the cell suspension was placed on a microscope slide and spread out with a coverslip. To observe redistribution of the cell-surface antigens, the slides were allowed to reach room temperature for about 30 min. This procedure leads to optimal capping (Hilgers *et al.*, 1975*a*). GAR/FITC and SWAM/

FITC were purchased from Nordic Laboratories (Tilburg, The Netherlands). Both conjugates reacted with gammaglobulins only, and contained about 2–3 mg protein/ml of specific IgG antibodies. The cells were examined with a Zeiss Fluorescence Microscope as described by Hilgers *et al.* (1975*a*). Cells were considered to show capping when antigen-associated FITC conjugate was redistributed into patches which occupied less than 50% of the cell surface and were asymmetrically distributed around the cell.

### RESULTS

### *Membrane fluidity of primary and transplanted GRSL cells*

The Table lists, in a condensed form, the P values measured at 25°C for various primary GRSLs and i.p. transplanted ascitic cell lines derived therefrom. The primary leukaemia cells and very early transplant generations ($\leqslant 3$) show significantly higher P values than do the later passages. This phenomenon is illustrated in Fig. 1 for individual cells of the GRSL16 cell line as compared with thymocytes. Individual cells of a given population exhibit marked differences in membrane fluidity which are not caused by their position in the cell-cycle (Tulp and Bont, 1975; Tulp *et al.*, 1977). The P values are symmetrically distributed and the downward shift in P value of the GRSL cells occurring on transplantation (from Passages 3 to 5) is not accompanied by the emergence of distinct subpopulations.

### *Capping of surface antigens in relation to membrane fluidity*

Fig. 2 illustrates the capping of virus-related (MLr, $G_{IX}$) and alloantigens (H-2.7(G), H-2.8(K) and TL1.2) as measured by the MIF test, in relation to P values of the cells. The measurements were performed on thymocytes, early- and late-passage GRSL cells and GRSL cells grown in solid form, s.c. or in the peritoneum wall. In all cases an identical correlation was found. Capping percentages of 50–100% were observed for P values >0·220 (demonstrated by thymocytes, very early transplant generations of GRSL ascitic cells and GRSL cells grown in solid form) whereas below that value (later passages of i.p. transplanted GRSL cells) the percentage of capped cells dropped considerably, being lowest at P values of 0·200–0·210. A representative example of extremely high capping of the viral-related MLr antigen is shown in Fig. 3.

### *Membrane fluidity of GRSL cells grown in immunologically crippled hosts*

A possible reason for the drop in P value occurring on transplantation of GRSL cells in intact GR mice follows from the results illustrated in Fig. 4, using the GRSL18 line. It is shown that the P

TABLE.—*Degrees of Fluorescence Polarization (P) at 25°C of Thymocytes, Primary and i.p. Transplanted GRSL Cells*

| Passage | GRSL16t | GRSL16s | GRSL18 | GRSL19 |
|---|---|---|---|---|
| 0 | | | 0·265 | 0·248 |
| 1 | 0·261 | 0·266 | 0·266 | 0·203 |
| 2 | 0·252 | | 0·190 | 0·210 |
| 3 | 0·229, 0·254 | 0·266 | 0·234 | 0·219 |
| 4 | 0·210 | 0·190, 0·191 | 0·224 | |
| 5 | 0·214 | 0·214, 0·228 | 0·223 | |
| 6–7 | | 0·202 ± 0·016 (3) | 0·226 | |
| 8–10 | 0·214 ± 0·013 (3)* | 0·178 ± 0·020 (4) | 0·222 ± 0·010 (4) | |
| 10–50 | 0·233 ± 0·019 (4) | | | |
| 10–50 | GRSL13 0·204 ± 0·026 (10) | | | |
| >50 | GRSL2 0·208 ± 0·020 (15) | | | |
| >50 | GRSL6 0·191 ± 0·017 (7) | | | |
| >50 | GRSL6a 0·209 ± 0·022 (14) | | | |
| — | Thymocytes 0·270 ± 0·010 (4) | | | |

* Mean values ± s.d. from different cell batches (numbers in parentheses).

FIG. 1.—Fluorescence polarization (P) values at 25°C for individual cells. Each symbol represents the value of a single cell. P was measured with the Elscint Single Cell Microviscosimeter. For labelling, cells were incubated with a $2 \times 10^{-5}$M DPH dispersion in PBS at 37°C for 15 min.

values of these cells remain essentially unchanged for 9 successive transplantation generations after the spontaneous origin, if transplanted into syngeneic mice irradiated by 400 rad whole body irradiation 24 h earlier. Such cells show capping percentages for the various antigens similar to those of primary GRSL cells. Finally, the scheme in Fig. 5 summarizes the range in P values found in normal thymocytes, primary GRSL cells, their ascitic transplants in normal and irradiated mice, and solid transplants resulting from s.c. transplantation or the occasional infiltration of ascitic cells into the peritoneal wall. Transplantation of ascitic cells from irradiated hosts into normal hosts is accompanied by a fall in P value, but the reverse shows a significant rise in only a few cases. Yet it is shown that adaptation of P values to environmental conditions of growth does take place since GRSL ascitic cells induced to grow in solid form show P values corresponding to those of the primary leukaemia cells. In this sense the solid form of growth (as it also occurs in primary leukaemias in thymus and spleen) correlates with high P values.

### DISCUSSION

The fluorescence polarization technique using DPH is now frequently used as a probe for measuring membrane lipid fluidity in intact cells, isolated plasma membranes, purified enveloped virus (Barenholz, Moore and Wagner, 1976) and lipid liposomes (Shinitzky and Inbar, 1974; Van Blitterswijk *et al.*, 1977; Barenholz *et al.*, 1976; Collard *et al.*, 1977). The degree of fluorescence polarization of DPH is related to the microviscosity of the lipid domain in which the probe is embedded, by the Perrin equation, as modified for rotational depolarization of a non-spherical fluorophore (Shinitzky *et al.*, 1971). High P values represent low lipid fluidity (*i.e.* high microviscosity) and *vice versa*.

As regards the significance of P values determined in intact cells, it was previously demonstrated for various cell systems, including the present one, that the method monitors lipids only. The difference in P values between intact thymocytes (P = 0·270) and transplanted (late-passage) GRSL ascitic cells (P = 0·208) was also exhibited by the corresponding isolated plasma membranes (P = 0·325 and 0·261, respectively) although the absolute values were lower for intact cells (Van Blitterswijk *et al.*, 1977). Similar results have been obtained on resting and regenerating rat-liver cells

FIG. 2.—Percentage of cells showing capping in the indirect membrane immunofluorescence (MIF) test for various cell-surface antigens, as a function of fluorescence polarization (P) at 25°C. Symbols: ○ and □: thymocytes; ◐ and ◧: early-passage (≤3) GRSL cells; ●: later passage GRSL cells; s: solid tumour (transplanted s.c. or grown on the peritoneal wall).

(Collard *et al.*, 1977). In these cell systems the difference in P values between intact cells and purified plasma membranes appeared to be predominantly due to probe signals stemming from the intracellular membranes of intact cells, into which DPH is apparently capable of rapid penetration (Van Blitterswijk *et al.*, 1977). However, it follows that differences in P values between whole cells of a comparative system reflect differences in cell-surface membrane fluidity.

The P value of a cell population, as listed in the Table, represents a mean value, while individual cells of that population may show a rather wide variation (Fig. 1). This may be the reason why a relation is demonstrable between mean P value and the percentage of capped cells (Fig. 2) if only the more rigid cells in the population are capable of capping. In addition, variation in concentration of antigen on the surface of individual cells could be involved. Although antigenic modulation *in vivo* leading to a release of MLr antigen-antibody complexes from the cell surface (Calafat *et al.*, 1976) may be instrumental in decreasing the viral antigenic content, this process cannot operate in the case of the normal antigens. Our results therefore suggest that, under the present experimental conditions, capping of surface antigens is favoured by increased membrane viscosity.

This correlation holds for thymocytes and GRSL cells and for both virus-related (MLr, $G_{IX}$) and normal (TL, H-2) cell-surface antigens. An analogous correlation has recently been established by Ben-Bassat *et al.* (1977), who showed that concanavalin A receptors on the surface of normal human lymphocytes exhibit a higher mobility than those on the more fluid surfaces of human lymphoma and leukaemia cells. However, it should be pointed out that the mouse GRSL cells used in the present study acquire a decreased fluidity and a decreased capping capacity only after one or few transplantations in the ascitic form. Primary and very early ascitic transplant generations or GRSL cells growing in solid form retain

FIG. 3.—Capping of MLr antigen on GRSL cells as seen in the indirect membrane immunofluorescence (MIF) test.

the characteristics of their normal homologues, the thymocytes.

The mechanism by which membrane rigidity favours capping of surface proteins remains to be established. An attractive possibility is the "squeezing out" of the membrane matrix of protein or protein–lipid complex, leading to a more peripheral position (Shinitzky and Inbar, 1976; Borochov and Shinitzky, 1976). However, the mechanism of capping is complex and it cannot be excluded that the observed correlation is an indirect rather than a direct one.

Since separate GRSL cells show individually different fluidities, a cell population with a different mean fluidity may emerge *in vivo* if a host selection mechanism existed that was somehow geared to a cell-surface property related to membrane fluidity. It is suggested from the results illustrated in Fig. 4 that the host immune response could be such a mechanism. Three recent findings support the existence of an immune response in GRSL-bearing GR mice. First antigen-antibody complexes are present in the ascitic fluid (Van Blitterswijk *et al.*, 1975). Second, humoral immunity to mammary-tumour virus, as measured by the sensitive radioimmunoprecipitation test (Ihle, Arthur and Fine, 1976) develops within 4 days after transplantation of GRSL cells into GR mice (Arthur, Hilgers and Fine, unpublished). Finally, augmented natural cytotoxicity of spleen lymphocytes, as described by

Herberman *et al.* (1977), occurs in GR mice with a peak around the fourth day after transplantation of GRSL cells (Spits

FIG. 4.—Fluorescence polarization (P) values of DPH at 25 °C in GR thymocytes, spontaneous thymus-derived (primary) leukaemia cells (GRSL18) and their ascites transplant generations in normal (● — ●) and irradiated (○ — ○) hosts. 400 rad whole-body irradiation was carried out 24 h before inoculation of the cells. The numbers in the Fig. indicate the number of days between i.p. inoculation ($10^7$ cells) and harvesting ($3–6 \times 10^8$ cells) when the mice were moribund. The age of the GR hosts ranged from 5 to 7 weeks.

and Hilgers, unpublished). If the host immune response causes the shift towards a more fluid cell population on transplantation, it apparently does so by eliminating the more rigid cells. Immune sensitivity and membrane rigidity might be related through the squeezing out of antigen in rigid lipid domains as mentioned above, leading to a greater antigenic expression or to an increased immunological reaction (Humphries and McConnell, 1975).

Finally, previous experiments have shown that transplanted GRSL cells exfoliate membranous vesicles of high rigidity into the ascitic fluid *in vivo* (Van Blitterswijk *et al.*, 1977). This may constitute another mechanism by which those cells acquire a high membrane fluidity. Although exfoliation of rigid membrane domains could be a more general process, its mediation by the immune response is feasible and is being studied.

We thank Dr Inbar for valuable discussions, Dr R. van Nie and Dr M. van der Valk for the search and histological diagnosis of primary GR strain leukaemias, and Elscint Ltd (in the person of Dr A. Bruck) for the opportunity to make use of their experimental model of the Single Cell Microviscosimeter.

In part supported by a contract with NCI (USA): NO1-CP-33368.

FIG. 5.—Schematic presentation of fluorescence polarization (P) values of GR thymocytes, primary thymus-derived leukaemias (GRSL), transplanted ascitic cells in normal and in irradiated hosts, and GRSL cells growing in solid form either in the peritoneal wall or s.c. Arrows denote changes in growth conditions. The height of the boxes denotes the variation in P values (= 2 × s.d.).

### REFERENCES

Barenholz, Y., Moore, N. F. & Wagner, R. R. (1976) Enveloped Viruses as Model Membrane Systems: Microviscosity of Vesicular Stomatitis Virus and Host Cell Membranes. *Biochemistry*, **15**, 3563.

Ben-Bassat, H., Polliak, A., Rosenbaum, S. M., Naparstek, E., Shouval, D. & Inbar, M. (1977) Fluidity of Membrane Lipids and Lateral Mobility of Concanavalin A Receptors in the Cell Surface of Normal Lymphocytes and Lymphocytes from Patients with Malignant Lymphomas and Leukemias. *Cancer Res.*, **37**, 1307.

Borochov, H. & Shinitzky, M. (1976) Vertical Displacement of Membrane Proteins Mediated by Changes in Microviscosity. *Proc. natn. Acad. Sci. U.S.A.*, **73**, 4526.

Calafat, J., Hilgers, J., Van Blitterswijk, W. J., Verbeet, M. & Hageman, P. C. (1976) Antibody-induced Modulation and Shedding of Mammary Tumor Virus Antigens on the Surfaces of GR Ascites Leukemia Cells as Compared with Normal Antigens. *J. natn. Cancer Inst.*, **37**, 1307.

Collard, J. G., De Wildt, A., Oomen-Meulemans, E. P. M., Smeekens, J., Emmelot, P. & Inbar, M. (1977) Increase in Fluidity of Membrane Lipids in Lymphocytes, Fibroblasts and Liver Cells Stimulated for Growth. *FEBS Lett.*, **77**, 173.

David, C. S., Schwartz, B. D., Okuda, K., Cullen, S. E., Hilgers, J. & Schwartz, R. H. Expression of Ia Antigens of T Cell Leukemias and Thymocytes. In *Proc. 3rd Ir Gene Workshop*. Ed. H. O. McDevitt. New York and London: Academic Press. (In press).

Herberman, R. B., Nunn, M. E., Holden, H. T., Staal, S. & Djeu, J. Y. (1977) Augmentation of Natural Cytotoxic Reactivity of Mouse Lymphoid Cells against Syngeneic and Allogeneic Target Cells. *Int. J. Cancer*, **19**, 555.

Hilgers, J. & Bentvelzen, P. (1978) Interaction between Viral and Genetic Factors in Murine Mammary Cancer. *Adv. Cancer Res.*, **26**, 143.

Hilgers, J., Van Blitterswijk, W. J., Bont, W. S., Theuns, G. J., Nusse, R., Haverman, J. & Emmelot, P. (1975*a*) Distribution and Antibody-induced Redistribution of a Mammary Tumor Virus-induced and a Normal Antigen on the Surface of Mouse Leukemia Cells. *J. natn. Cancer Inst.*, **54**, 1335.

Hilgers, J., Haverman, J., Nusse, R., Van Blitterswijk, W. J., Cleton, F. J., Hageman, Ph. C., Van Nie, R. & Calafat, J. (1975*b*) Immunology, Virologic, and Genetic Aspects of Mammary Tumor Virus-induced Cell Surface Antigens: Presence of these Antigens and the Thy1.2 Antigen on Murine Mammary Gland Tumor Cells. *J. natn. Cancer Inst.*, **54**, 1323.

Humphries, G. M. K. & McConnell, H. M. (1975) Antigen Mobility in Membranes and Complement-mediated Immune Attack. *Proc. natn. Acad. Sci. U.S.A.*, **72**, 2483.

Ihle, J. N., Arthur, L. O. & Fine, D. L. (1976) Autogenous Immunity to Mouse Mammary Tumor Virus in Mouse Strains of High and Low Mammary Tumor Incidence. *Cancer Res.*, **36**, 2840.

Inbar, M. (1976) Fluidity of Membrane Lipids: a Single Cell Analysis of Mouse Normal Lymphocytes and Malignant Lymphoma Cells. *FEBS Lett.*, **67**, 180.

Inbar, M., Shinitzky, M. & Sachs, L. (1974) Microviscosity in the Surface membrane Lipid Layer of Intact Normal Lymphocytes and Leukemic Cells. *FEBS Lett.*, **38**, 268.

Nicolson, G. L. (1976*a*) Trans-membrane Control of the Receptors on Normal and Tumor Cells. I. Cytoplasmic Influence over Cell Surface Components. *Biochim. biophys. Acta*, **457**, 57.

Nicolson, G. L. (1976*b*) Trans-membrane Control of the Receptors on Normal and Tumor Cells. II. Surface Changes Associated with Transformation and Malignancy. *Biochim. biophys. Acta*, **458**, 1.

Shinitzky, M., Dianoux, A. C., Gitter, C. & Weber, G. (1971) Microviscosity and Order in the Hydrocarbon Region of Micelles and Membranes Determined with Fluorescent Probes. I. Synthetic Micelles. *Biochemistry*, **10**, 2106.

Shinitzky, M. & Inbar, M. (1974) Difference in Microviscosity Induced by Different Cholesterol Levels in the Surface Membrane Lipid Bilayer of Normal Lymphocytes and Malignant Lymphoma Cells. *J. mol. Biol.*, **85**, 603.

Shinitzky, M. & Inbar, M. (1976) Microviscosity Parameters and Protein Mobility in Biological Membranes. *Biochim. biophys. Acta*, **433**, 133.

Singer, S. J. & Nicolson, G. L. (1972) The Fluid Mosaic Model of the Structure of Cell Membranes. *Science, N.Y.*, **175**, 720.

Staats, J. (1976) Standardized Nomenclature for Inbred Strains of Mice: 6th Listing. *Cancer Res.*, **36**, 4333.

Stockert, E., Old, L. J. & Boyse, E. A. (1971) The $G_{IX}$ System. A Cell Surface Alloantigen Associated with Murine Leukemia Virus: Implications Regarding Chromosomal Integration of the Viral Genome. *J. exp. Med.*, **133**, 1334.

Tulp, A. & Bont, W. S. (1975) An Improved Method for the Separation of Cells by Sedimentation at Unit Gravity. *Analyt. Biochem.*, **67**, 11.

Tulp, A., Welagen, J. J. M. N., Van Blitterswijk, W. J. & Sluyser, M. (1977) Separation of Mammalian Cells and Nuclei by Velocity Sedimentation at Ambient Gravity. In *Proc. 3rd Int. Symp. Pulse Cytophotometry*. Ed. C. A. M. Haanen, H. F. P. Hillen and J. M. C. Wessels. Ghent: European Press. (In press).

Van Blitterswijk, W. J., Emmelot, P., Hilgers, J., Kamlag, D., Nusse, R. & Feltkamp, C. (1975) Quantitation of Virus-induced (MLr) and Normal (Thy1.2) Cell Surface Antigens in Isolated Plasma Membranes and the Extracellular Ascites Fluid of Mouse Leukemia Cells. *Cancer Res.*, **35**, 2743.

Van Blitterswijk, W. J., Emmelot, P., Hilkmann, H. A. M., Oomen-Meulemans, E. P. M. & Inbar, M. (1977) Differences in Lipid Fluidity among Isolated Plasma Membranes of Normal and Leukemic Lymphocytes and Membranes Exfoliated from their Cell Surface. *Biochem. biophys. Acta*, **467**, 309.

Zachařová, C., Renčková, L., Dux, A. & Démant, P. (1975) I Region Associated Histoincompatibility with Absence of Accelerated Rejection of Second Set Skin Grafts Detected in Tests with New Haplotype, H-$2^{dx}$. *J. Immunogenetics*, **2**, 323.